# Bony Path Assisted Suturing and Tendon-to-Tendon Suturing Technique for External Rotator Repair After Hip Arthroplasty: A Randomized-Controlled Trial

**DOI:** 10.1007/s43465-026-01748-8

**Published:** 2026-04-21

**Authors:** Zuzhou Wen, Yun Ye, Hongqiang Chen, Junzhao Qiao, Xizhi Wu, Ang Luo

**Affiliations:** Department of Joint Orthopedics, The Fourth People’s Hospital of Guiyang, Guiyang, Guizhou China

**Keywords:** Total hip arthroplasty, Suturing techniques, External rotator muscle repair, Randomized-controlled trial

## Abstract

**Objective:**

To compare and verify the effectiveness of tendon-to-bone suture technique for the repair of external rotator injuries after hip arthroplasty.

**Methods:**

This was a prospective, randomized, controlled study including 178 patients that were divided into two groups. Clinical outcomes, joint dislocation rates, and external rotation angles were compared between patients who underwent the tendon-to-bone suture technique (group I) and those who underwent the tendon-to-tendon suture technique (group II).

**Results:**

There were no statistically significant differences between the two groups in terms of sex, age, operative side, body mass index, diagnosis, visual analog scale (VAS) score, operation time, blood loss, or incision length (*P* > 0.05). The hip Harris scores of the two groups were significantly higher in group I than in group II at 1 and 3 months after surgery (*P* < 0.05); however, the difference was not statistically significant at 6 months after surgery (*P* > 0.05). The difference in postoperative VAS scores of the two groups was not statistically significant (*P* > 0.05). The incidence of postoperative joint dislocation in group I was significantly lower than in group II (*P* < 0.05). The external rotation angles of the two groups were higher in group I than in group II at 1, 3, and 6 months after surgery (*P* < 0.05); however, after 12 months of rehabilitation, the external rotation angles were close to normal, and the difference between the two groups was not statistically significant (*P* > 0.05).

**Conclusion:**

Tendon-to-bone repair is superior to tendon-to-tendon repair. Crucially, this technique enhances therapeutic outcomes without increasing operative time, incision length, or blood loss.

## Introduction

Total hip arthroplasty (THA) is an effective method for treating hip diseases and reconstructing hip function, and can effectively relieve hip pain, significantly improve function, and enhance quality of life [1]. Prosthesis dislocation is a common complication of THA, accounting for approximately 2.5% of cases, with a significant impact on long-term prosthesis survival; furthermore, 11–24% of hip revisions are secondary to recurrent dislocations [2]. Most dislocations occur in the early postoperative period, with 75% accounting for posterior dislocations [3], the causes of which include poor prosthesis position, bony impingement, weak abductors, and neurological disorders [4]. Most current studies have focused on prosthesis position, which has led to an underestimation of the importance of external rotators in ensuring the stability of the hip joint in the postoperative period. It has been reported that external rotator muscle groups play an important role in maintaining the head-socket alignment of the hip joint, the balance of muscle strength, and the stability of the line of force of the movement of the center of rotation of the hip joint [5], and in preventing the occurrence of posterior hip dislocation [6].

Several studies have confirmed that posterior lateral THA causes damage to the external rotator muscle [7–9]. Currently, the widely used clinical method for repairing hip external rotator injuries primarily involves direct suturing between the torn soft-tissue ends. This surgical approach, to some extent, aligns with the principle of anatomical reconstruction and helps restore the local biomechanical structure of the joint. However, despite its theoretical advantage in structural restoration, existing clinical data indicate a high rate of repair failure following direct suture surgery, including issues such as retearing, poor healing, and suboptimal functional recovery [10–13].

Therefore, this paper describes the application of tendon-to-bone suture technique for the repair of the external rotators and joint capsule after THA, with the aim of exploring whether the tendon-to-bone suture technique provides better clinical outcomes compared with the tendon-to-tendon suture technique, particularly in terms of joint dislocation rates and abductor muscle strength.

## Materials and Methods

This prospective study was approved by the hospital ethical review board and was registered with the International Traditional Medicine Clinical Trial registry. Furthermore, the study was reported in accordance with the rigor of the CONSORT guideline [14], and all experimental conditions conformed to the Declaration of Helsinki. All patients or their families signed an informed consent form.

The inclusion criteria comprised patients admitted with a diagnosis of unilateral hip disease undergoing primary hip arthroplasty, encompassing specific conditions such as hip dysplasia (Crowe Type I or II), avascular necrosis of the femoral head, or primary hip osteoarthritis. Exclusion criteria encompass prior surgery on the same hip, concomitant femoral neck or intertrochanteric fractures, patients requiring hip revision surgery (e.g., due to infection, prosthesis loosening, periprosthetic fractures, or bone defects), use of cemented prostheses or specialized prostheses (e.g., 3D-printed prostheses, tumor prostheses, distal fixation prostheses, or modular prostheses), joint ankylosis or rigidity, severe developmental dysplasia of the hip (Crowe III or IV), pathological or malignant hip disease, septic or rheumatoid hip arthritis, severe myasthenia gravis, muscular atrophy, or other neurological conditions associated with muscle wasting.

To eliminate any possible age differences, we compared patients treated with the tendon-to-bone suture technique with those treated with the tendon-to-tendon suture technique at different ages (< 60 years and > 60 years).

Previous studies have shown that when using the posterolateral approach for THA, the failure rate of transosseous soft-tissue repair for the short external rotator muscles can be as low as 20% [15]. Biomechanical studies have indicated that the stability of tendon-to-bone repair is twice that of tendon-to-tendon repair [16]. Based on this estimate, the present study conducted a priori power analysis. With α set at 0.05 and a test power of 0.8, at least 82 hip joint samples per group were required. Thanks very much again.

### Randomization and Blinding

**Fig. 1:**
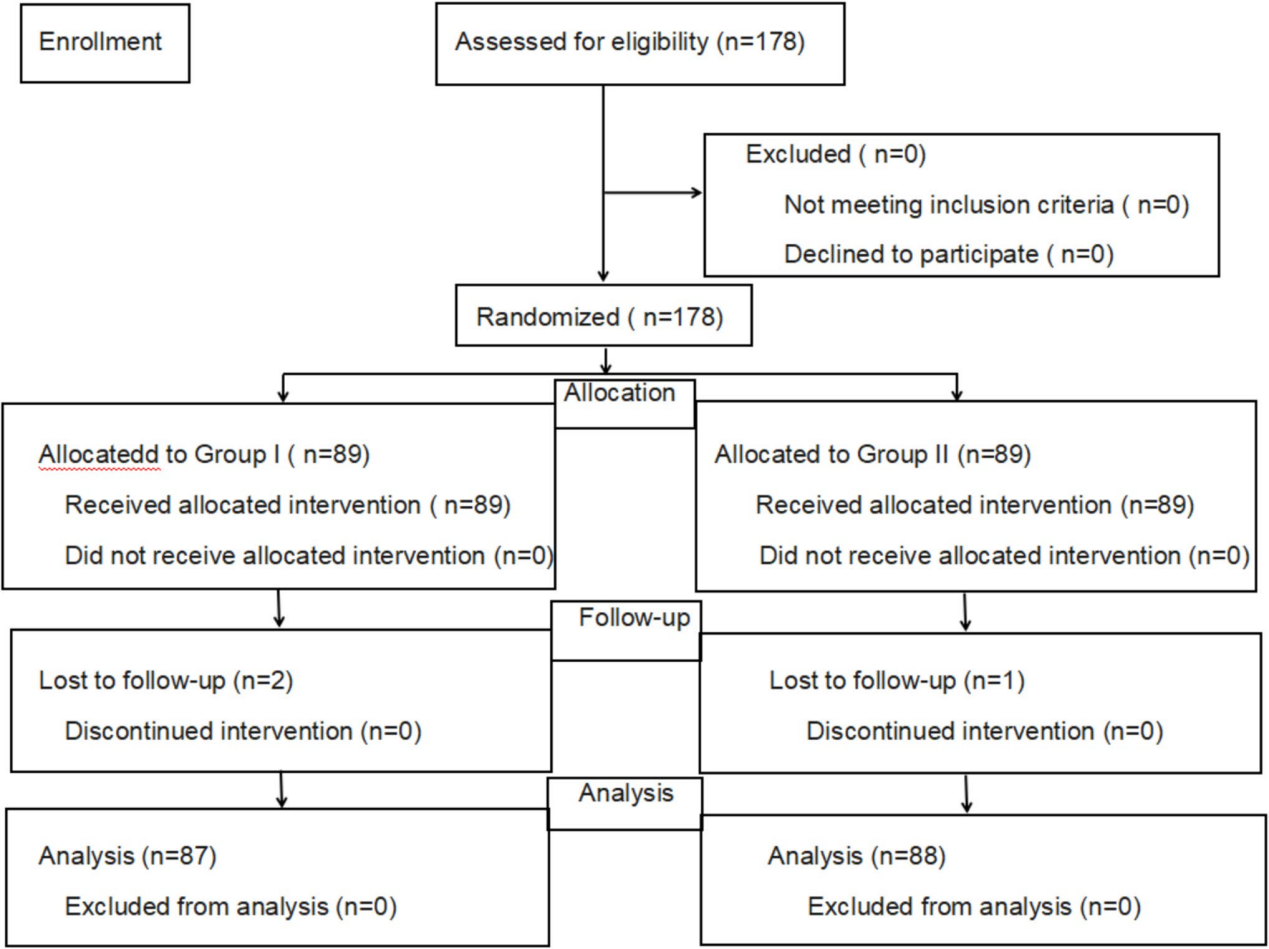
Consolidated standards of reporting trials flow diagram showing the progress of patients through the study

A total of 178 patients were recruited; patients were randomly divided into two groups: group I and group II in a 1:1 ratio according to a computer-generated randomization sequence. The random sequence was generated with sequentially numbered, opaque, and sealed envelopes by surgeons. Before the commencement of the surgery, the physician randomly selected a number, that is, tendon-to-bone suture technique for group I and tendon-to-tendon suture technique for group II. The patients, surgeons, and data collectors were all unaware of the group assignment (Fig. 1).

### Surgical Procedures

Patients in each of the two groups were randomized by a single surgeon. In the healthy-side lying position, the surgery was performed via the conventional posterolateral approach, extending 5.0 cm upward from the top of the greater trochanter along the line of the posterior superior iliac spine and 4.0 cm downward; an incision was made through the skin, subcutaneous tissues, and tensor fascia lata; and the externally rotated muscle groups (Piriformis, Superior Gemellus, Obturator Internus, and Inferior Gemellus) were severed, to expose the posterior joint capsule, employ a standard inverted T-shaped incision to dissect the joint capsule, taking care to preserve its integrity. The femoral head was obliquely osteotomized along the inter-rotor line at 1.0–1.5 cm above the lesser trochanter, the femoral head was removed posteriorly, the acetabular labrum and intra-acetabular fat were removed, the acetabulum was polished with an acetabular file until there was dense pitting of the hemorrhage, and part of the femoral head was implanted in the bottom of the acetabulum with the cancellous bone and bone clay. Subsequently, the acetabular cup and liner were placed. The proximal femur was expanded in the usual manner, the appropriate type of femoral stem was inserted (keeping the natural anterior inclination of the femur), the ball head was installed, and the hip joint was reset and checked for stability. In all patients, we performed the Shuck Test, axial traction on the femur to assess soft-tissue tension, with 2–4 mm of traction, demonstrating adequate tension to prevent subluxation. In the ROM test, flex the hip to 90°, adduct, and internally rotate (the ‘position of risk’ for posterior dislocation). Combined anteversion assessment, using the Ranawat sign: with the knee flexed 90°, the femoral component’s axis should align with the tibia’s axis when combined anteversion is optimal; none of the patients experienced dislocation. Finally, the joint capsule is sutured in situ to restore its anatomic structure and physiological function.

**Fig. 2:**
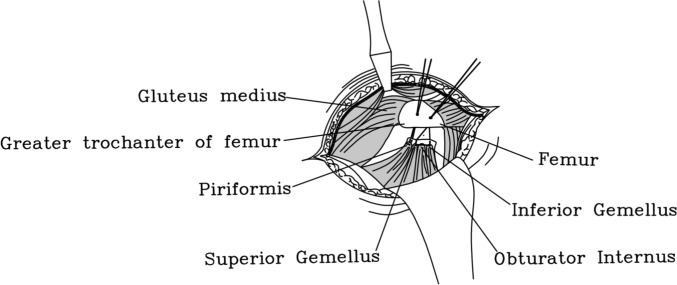
Anatomical diagram of tendon-to-bone suture technique

**Fig. 3:**
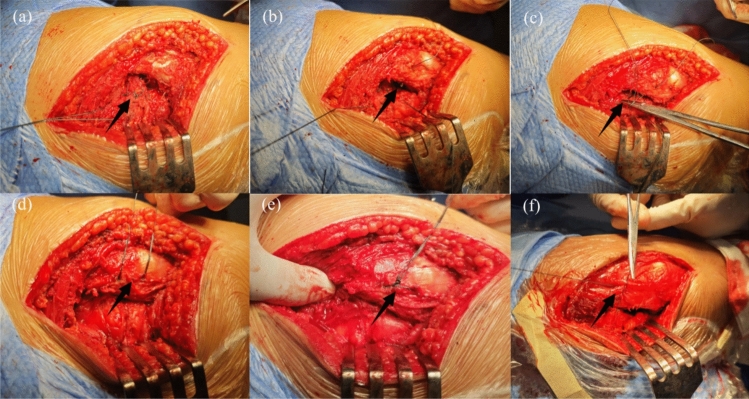
**a** Systematically suture the piriformis muscle and the remaining short external rotator muscles (Superior Gemellus, Obturator Internus, and Inferior Gemellus) using size 1 absorbable surgical suture. **b** A secondary threading operation is performed with an absorbable suture of the same type through the prefabricated osseous tunnel. **c** The end of the suture that has already been sutured to the external rotator muscle group is retrograded through the osseous tunnel, with the assistance of a needle holder. **d** Appropriate tension is applied to achieve mechanical equilibrium within the bony tunnel. **e** A standard surgical knot (square knot overlaid with a slip knot) is used to complete the fixation to ensure a secure ligation. **f** Employed size 1 absorbable surgical suture to perform in situ tendon-to-tendon suturing of the external rotator muscle

**Fig. 4:**
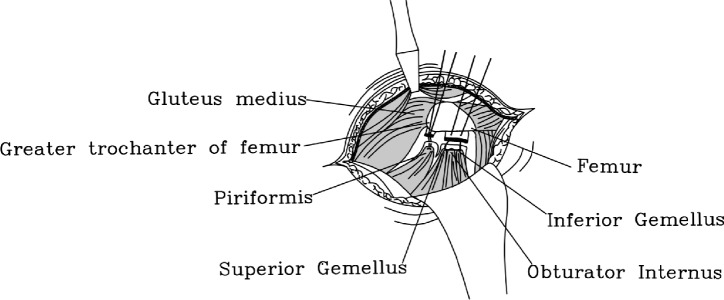
Anatomical diagram of tendon-to-tendon suture technique

Next, we will proceed with the reconstruction of the short external rotators. For Group I patients, two bony tunnels are created using 2.0 mm metal bone pins at the posterior aspect of the greater trochanter of the femur, along the attachment points of the external rotators, from outside to inside. Subsequently, using size 1 absorbable surgical suture, the short external rotator muscle group is reconstructed non-in-situ via the bony tunnels posterior to the greater trochanter, employing the tendon-to-bone (Fig. 2) technique. First, systematically suture the piriformis muscle and the remaining short external rotator muscles (Superior Gemellus, Obturator Internus, and Inferior Gemellus) using size 1 absorbable surgical suture. Second, a secondary threading operation is performed with an absorbable suture of the same type through the prefabricated osseous tunnel; the end of the suture that has already been sutured to the external rotator muscle group is retrograded through the osseous tunnel, with the assistance of a needle holder. Appropriate tension is applied to achieve mechanical equilibrium within the bony tunnel. Finally, a standard surgical knot (square knot overlaid with a slip knot) is used to complete the fixation to ensure a secure ligation (Fig. 3). In group II, we preserved some of the external rotator stops during the stripping process, and after the procedure was completed, we employed size 1 absorbable surgical suture to perform in situ tendon-to-tendon (Fig. 4) suturing of the external rotator muscle (Fig. 3).

In all our patients, the acetabular cup was positioned within the safe range of anteversion (10°–25°) during surgery, with the combined anteversion set between 25° and 50°. Regarding implant types, acetabular liners were uniformly constructed from high-molecular-weight polyethylene. High-rim liners were consistently positioned posterosuperiorly (approximately at the 11 o'clock position). The femoral heads were ceramic and of standard size. Patients who underwent intraoperative head-neck adjustments, had abnormal femoral offset (either too large or too small), or had abnormal femoral neck length (either too long or too short) were excluded postoperatively. Postoperative CT scans were used to measure all cases, and individuals with anteversion outside the reasonable range were further excluded.

### Postoperative Treatment

All patients were administered prophylactic antibiotics to prevent infection and anticoagulants to prevent deep vein thrombosis after surgery. Joint functional rehabilitation exercises (quadriceps training, ankle pump, gluteal extension, adductor extension, knee flexion with bed/chair support, knee flexion with seated support, and knee extension with unsupported sitting support) were initiated on the first postoperative day for each patient.

### Clinical Outcome Assessment

All demographic data and patient-reported outcomes were prospectively collected by the same surgeon, with preoperative, 1-, 3-, and 6-month postoperative Harris scores of the hip, and postoperative external rotation angles of the hip at 1, 3, 6, and 12 months after surgery, who had no knowledge of the surgical procedure or suture modalities. Baseline data on sex (male/female), age at the time of surgery (years), laterality (left/right), body mass index (BMI; kg/m^2^), diagnosis [developmental dysplasia of the hip (DDH)/osteonecrosis of the femoral head (ONFH)/osteoarthritis (OA)], operative time (min), blood loss (ml), and incision length (cm) were collected. The main indexes evaluated were the postoperative hip Harris score and visual analog scale (VAS) score (mean ± standard deviation), joint dislocation rate, and hip external rotation range measurement (°).

### Statistical Analysis

All statistical analyses were performed using SPSS software (version 26; SPSS Inc., Chicago, IL, USA). Independent *t* tests were performed for continuous variables that conformed to a normal distribution, and numerical variables were expressed as the mean ± standard deviation (SD). For categorical variables that did not show a normal distribution, the χ2 test was used for counting information, and when a *P* value of < 0.05 was considered to be statistically significant.

## Results

In group I and group II, two and one patient were lost to follow-up after the operation; therefore, 87 and 88 patients were finally included in each group, respectively.

The baseline characteristics of the patients in the two groups and comparison of the different age stages (< 60 years and > 60 years) showed no difference (*P* > 0.05; Table 1). At 1 and 3 months after surgery, the Harris scores of the affected hip joints of patients were significantly higher in group I (70.37 ± 6.90 and 85.16 ± 6.52) than in group II (64.84 ± 8.12 and 82.12 ± 6.13; *t* = 4.849, *P* < 0.05 and *t* = 3.020, *P* < 0.05, respectively). The difference between the Harris scores of the affected hip joints of the two groups at 6 months postoperatively was not statistically significant (*P* > 0.05), and the difference in the VAS scores of the two groups postoperatively was not statistically significant (*P* > 0.05; Table 2).

**Table 1:** Basic information (mean ± SD)

		Group I	Group II	Statistical value	*P* value
Sex	Male	42	41	0.050^a^	0.823
	Female	45	47		
Age (years)		64.23 ± 7.93	62.90 ± 8.07	1.101^b^	0.272
	< 60	34	40	0.728^a^	0.393
	> 60	53	48		
Side	Left	40	38	0.138^a^	0.710
	Right	47	50		
BMI (kg/m^2^)		23.40 ± 3.30	23.13 ± 3.35	0.552^b^	0.582
Diagnosis	DDH	21	27	1.461^a^	0.482
	ONFH	42	35		
	OA	24	26		
Operation time (min)		70.03 ± 12.85	68.36 ± 10.36	0.950^b^	0.344
Blood loss (ml)		195.63 ± 20.48	201.52 ± 23.46	− 1.769^b^	0.079
Incision length (cm)		13.61 ± 2.27	13.38 ± 2.13	0.704^b^	0.482

DDH, developmental dysplasia of the hip; ONFH, osteonecrosis of the femoral head; OA, osteoarthritis

^a^*χ*^2^value

^b^*t* value

**Table 2:** Harris score and VAS score (mean ± SD)

	Harris score (0–100)				VAS score (0–10)	
	Pre-operation	1 month	3 months	6 months	Pre-operation	Post-operation
Group I	38.57 ± 7.11	70.37 ± 6.90	85.16 ± 6.52	90.13 ± 5.11	8.26 ± 1.07	4.70 ± 1.09
Group II	36.67 ± 8.08	64.84 ± 8.12	82.12 ± 6.13	89.90 ± 5.12	8.35 ± 1.02	4.72 ± 0.99
*t* value	1.655	4.849	3.020	0.296	– 0.556	– 0.166
*P* value	0.10	0.000	0.003	0.768	0.579	0.868

### Joint Dislocation Rate

The 1-year postoperative joint dislocation rates in groups I and II were 0 and four cases, accounting for 0% and 4.5%, respectively, and the differences were statistically significant (*P* < 0.05; Table 3), suggesting that repairing the external rotator muscle after hip arthroplasty using the tendon-to-bone suture technique is advantageous for ensuring postoperative joint stability.

**Table 3:** Joint dislocation rate

	Number of joint dislocations	Number of non-dislocated joints	Total	Dislocation rate
Group I	0	87	87	0%
Group II	4	84	88	4.5%
total	4	171	175	2.3%
χ² value	4.047^a^			
*P* value	0.044			

“a” indicates the χ² value

### External Rotation Range

**Fig. 5:**
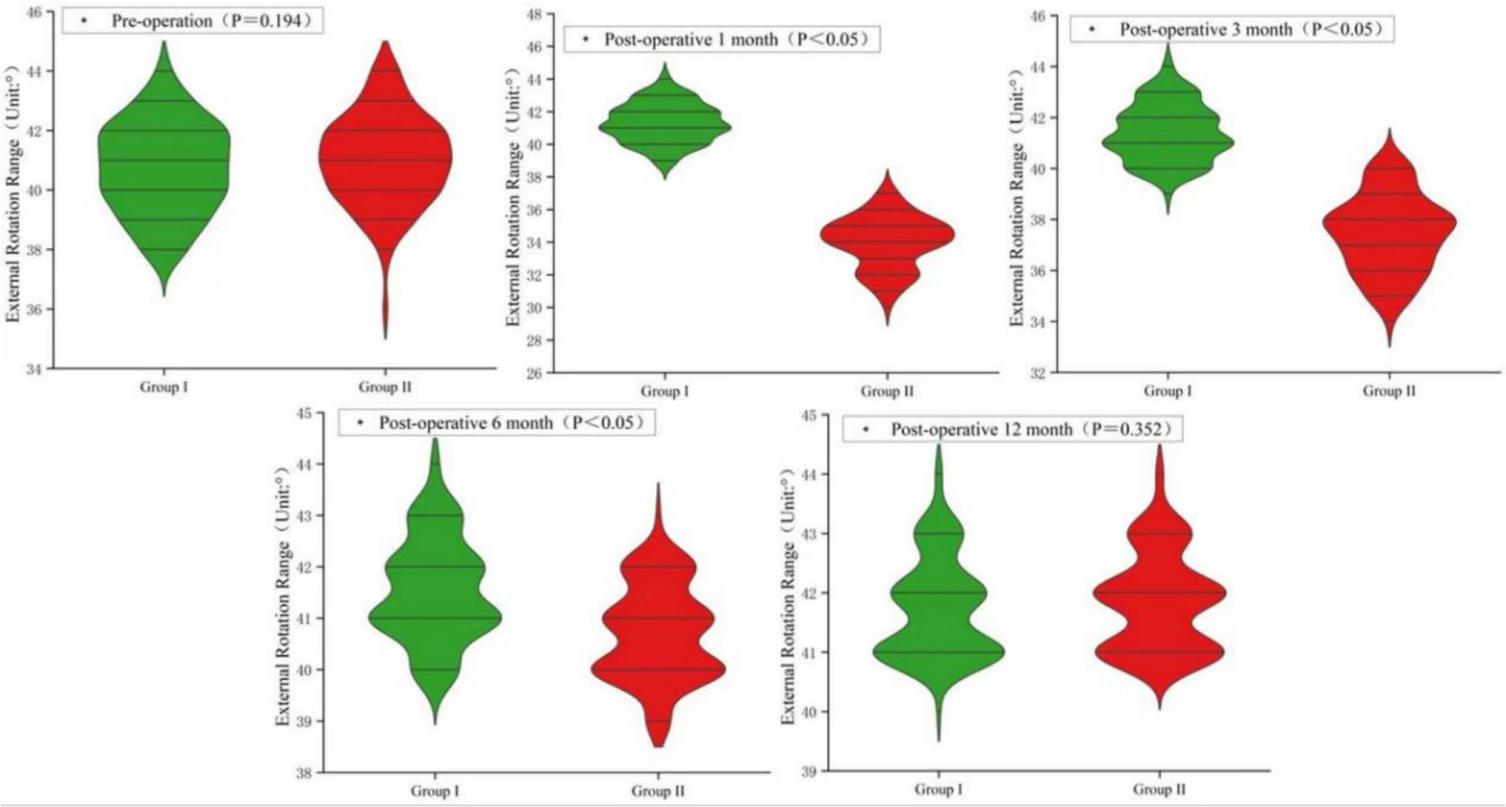
Comparison of hip external rotation angles between the tendon-to-bone suture technique (group I) and the tendon-to-tendon suture technique (group II) at 1, 3, 6, and 12 months after surgery. The external rotation angles of group I were higher than those of group II at 1, 3, and 6 months after surgery (*P* < 0.05); the external rotation angles at 12 months were close to normal, and the difference was not statistically significant between the two groups (*P* > 0.05)

The external rotation range of the hip joint of the affected limb at 1 and 3 months after surgery was significantly higher in group I (40.30° ± 1.19° and 41.40° ± 1.10°) than in group II (34.51° ± 1.59° and 37.44° ± 1.50°; *P* < 0.05); it was close to the normal value at 6 months after surgery, but still statistically significant. After 12 months of rehabilitation, the external rotation angle was close to normal, and the difference between the two groups was not statistically significant (*P* > 0.05; Fig. 5).

### Complications

No postoperative complications, such as wound infection, hematoma, deep vein thrombosis of the lower extremities, nerve injury, loosening of the prosthesis, or periprosthetic fracture, occurred in either group.

## Discussion

The number of hip disorders is increasing [17]. Approximately 240 million people worldwide suffer from OA of the hip, and approximately 10% of all patients over the age of 45 years suffer from imaging manifestations of OA of the hip [2, 18]. The prevalence of ischemic necrosis of the femoral head is two cases per 100,000 people and is more common in males, with the highest prevalence in males aged 25–44 years and females aged 55–75 years [19, 20]. The prevalence of hip dysplasia varies according to country and race, ranging from 5.4 to 12.8% in Denmark, 1.8% in Korea, 2.4% in Turkey, and 7.3% in Singapore [21]. THA is the most effective treatment option for end-stage lesions of the above diseases and can significantly alleviate the clinical symptoms of patients, restore the center of rotation of the hip joint, and simultaneously correct the biological lines of force of the hip joint to improve the quality of life of elderly patients [22].

The posterolateral approach is the most commonly used surgical approach for artificial THA [23] and has the advantages of clear anatomical layers, a low incidence of heterotopic ossification, and a short learning cycle. However, the extent of damage to the hip external rotator and the need for repair using this approach remain controversial. Researchers who support repair believe that the repaired external rotator muscle provides physical support similar to a brace, which stabilizes the hip joint and reduces the rate of prosthesis dislocation in the early postoperative period; therefore, effective repair is necessary [24–26]. More balanced and coordinated hip motion and better external rotation function have also been mentioned [27]. However, due to the significant risk of re-rupture and failure of posterior lateral access external rotator repairs, opponents of posterior lateral access external rotator repairs insist that the repaired external rotator does not meet the strength demands of daily activities, and some insist that the repairs themselves are not responsible for the lower dislocation rates [28, 29].

The results of this study demonstrate the importance of external rotator repair after artificial THA and that effective external rotator repair is beneficial for improving patients' postoperative joint function and the risk of dislocation. To address this issue, we advocate drilling two bony channels on the posterior aspect of the greater trochanter with a 2.0 mm metal bone pins, and then reconstructing the external rotator muscle group reconstructed using transosseous fixation through bony channels through the bony channels on the posterior aspect of the greater trochanter using a No. 1 absorbable surgical suture.

In this study, the Harris scores of the hip joints of the two groups of patients were significantly higher in group I than in group II at 1 and 3 months after surgery (*P* < 0.05), and the incidence of postoperative joint dislocation was significantly lower in group I than in group II (*P* < 0.05). Furthermore, the external rotation angle of both groups was higher in group I than in group II at 1, 3, and 6 months after surgery (*P* < 0.05), indicating that this tendon-to-bone suture technique applied to the repair of the external rotator muscle after THA is reliable. In addition, it can make the artificial hip joint closer to the physiological state of the human body to obtain a good soft-tissue balance, which plays an important role in the restoration of the patient's late hip joint function. Most surgeons use simple sutures for the external rotator muscle, which seriously underestimates the importance of the external rotator muscle in ensuring joint stability during the postoperative period.

### Limitations

This study has certain limitations, including its single-center nature and limited sample size. To more comprehensively validate and assess the safety of this technique, as well as to ensure the generalizability of the study results in different clinical settings, it is necessary to expand to multicenter randomized controls and incorporate larger sample sizes in the future to reduce the bias that may be brought about by a single center or a limited sample size and to increase the reliability and extrapolation.

## Conclusion

In conclusion, posterior lateral approach THA causes significant damage to the external rotator muscle group, whereas effective muscle repair facilitates early postoperative recovery in patients. This prospective, single-center, randomized controlled clinical trial assessed that tendon-to-bone repair provides superior stability compared to tendon-to-tendon repair. Posterior soft-tissue repair using tendon-to-bone technique demonstrated a lower dislocation rate than tendon-to-tendon repair. Crucially, this technique enhances therapeutic outcomes without increasing operative time, incision length, or blood loss.

## Data Availability

The datasets used and analyzed during the current study are available from the corresponding author on reasonable request.
